# Effects and long-term outcomes of endurance versus resistance training as an adjunct to standard medication in patients with stable COPD: a multicenter randomized trial

**DOI:** 10.1186/s12890-024-03010-z

**Published:** 2024-04-22

**Authors:** Shilei Cui, Haiying Ji, Li Li, Huili Zhu, Xiangyang Li, Ying Gong, Yuanlin Song, Lijuan Hu, Xu Wu

**Affiliations:** 1 Department of Pulmonary and Critical Care Medicine, Zhongshan Hospital, Fudan University, 180 Feng Lin Rd, Shanghai, 200032 China; 2 Department of Pulmonary and Critical Care Medicine, Huadong Hospital, Fudan University, Shanghai, 200040 China; 3 Shanghai Respiratory Research Institute, Shanghai, 200032 China; 4 National Clinical Research Center for Aging and Medicine, Huashan Hospital, Fudan University, Shanghai, 200032 China

**Keywords:** Chronic obstructive pulmonary disease, Pulmonary rehabilitation, Endurance training, Resistance training, Exercise capacity, Health-related quality of life

## Abstract

**Background:**

Comparisons between endurance training (ET) and resistance training (RT) have produced equivocal findings in chronic obstructive pulmonary disease (COPD) patients. The purpose of our study is to investigate the effectiveness and long-term outcomes of adding ET and RT to conventional medical treatment in patients with COPD. A secondary objective is to investigate the clinical improvements resulting from exercise training in patients with different disease severities.

**Methods:**

The study was a multicenter, prospective trial in people with stable COPD. The cohort was randomized to three groups: individualized medical treatment group (MT), MT + endurance training group (MT + ET) and MT + resistance training group (MT + RT). Exercise was performed 3 times weekly over a 12-week period. The endpoints of exercise capacity, health-related quality of life, COPD symptoms, lung function, and anxiety and depression questionnaires were re-evaluated at baseline, at the completion of the intervention and at 6 and 12-month follow-up. According to the COPD assessment tool offered by GOLD guidelines, patients were stratified into GOLD A and B groups and GOLD C and D groups for further subgroup analysis.

**Results:**

The intention-to-treat (ITT) population included 366 patients, 328 of them completed the study protocol over 12 months (the PP-population). There were no significant differences in the primary outcome, quality of life, between patients who underwent medical treatment (MT) alone, MT + endurance training (MT + ET), or MT + resistance training (MT + RT) at the completion of the intervention, 6-, or 12-month follow-up. Additionally, no significant differences were observed between MT, MT + RT, or MT + ET groups concerning the primary outcome, exercise capacity (3MWD), after initial 3 months of intervention. However, a small statistically significant difference was noted in favor of MT + ET compared to MT + RT at 12 months (ITT: Δ3MWD in ET vs RT = 5.53 m, 95% confidence interval: 0.87 to 13.84 m, *P* = 0.03) (PP: Δ3MWD in ET vs RT = 7.67 m, 95% confidence interval: 0.93 to 16.27 m, *P* = 0.04). For patients in the GOLD C and D groups, improvement in quality of life following ET or RT was significantly superior to medical intervention alone. Furthermore, upon completion of the exercise regimen, RT exhibited a greater improvement in anxiety compared to ET in these patients (ITT: ΔHAD-A at 3-month: RT = -1.63 ± 0.31 vs ET = -0.61 ± 0.33, *p* < 0.01) (PP: ΔHAD-A at 3-month: RT = -1.80 ± 0.36 vs ET = -0.75 ± 0.37, *p* < 0.01).

**Conclusions:**

Our study presents evidence of the beneficial effects of ET and RT in combination with standard medical treatment, as well as the long-term effects over time after the intervention. While the statistically significant effect favoring ET over RT in terms of exercise capacity was observed, it should be interpreted cautiously. Patients in severe stages of COPD may derive greater benefits from either ET or RT and should be encouraged accordingly. These findings have implications for exercise prescription in patients with COPD.

**Trial registration:**

ChiCTR-INR-16009892 (17, Nov, 2016).

## Background

With increasing social and economic burden, chronic obstructive pulmonary disease (COPD) contributes to substantial morbidity and has increased in rank from 11 to 6th in disability-adjusted life years (DALYs) among all causes between 1990 and 2019 [1]. Patients with COPD suffer from comorbidities such as cardiovascular disease and skeletal muscle dysfunction, and are at high risk of depression and anxiety, all of which could greatly reduce quality of life and impair exercise capacity of the patients during the course of the disease [2].

Pulmonary rehabilitation (PR) is a well-recognized, comprehensive intervention advocated for the management of COPD. As an essential component of pulmonary rehabilitation (PR), exercise training has demonstrated benefits in physical capacity, dyspnea, anxiety and depression in those afflicted with COPD [3]. The conventional modalities of exercise training are mainly described as endurance training (ET) and resistance training (RT) [4]. Progressive endurance training provides a training mode for increasing cardiorespiratory system function in PR programs. In addition, resistance training resulted in more improvements in peripheral muscle strength with less dyspnea during exercise [5]. Recent randomized trials comparing the effectiveness of ET and RT have reported conflicting conclusions [6]. Some studies support the superiority of RT over ET, while others suggest the opposite [7, 8]. In addition, some studies have suggested a combination of both, rather than one modality alone [9, 10]. It is uncertain whether there is an optimal exercise programme to achieve maximum benefit from treatment [11]. The reason for the inconsistent findings may be related to the different exercise intensity and exercise regimen settings in the study design. Meanwhile, patients commonly fail to attend or complete their course mainly because of skeletal muscle dysfunction [12] and symptoms of dyspnea [13], thus evoking a vicious cycle. While the guidelines suggest that patients at all stages of COPD can benefit from pulmonary rehabilitation, some studies suggest that patients may benefit differently from exercise training due to different physical abilities and medical conditions [14, 15]. A generalised exercise training program is clearly deficient in patients with different disease severities. Hence, identifying the optimal type of strategies that will yield the greatest improvements may promote the long-term adherence to exercise programs.

Although PR is a high-value treatment, some benefits tend to diminish following completion of the rehabilitation program [16]. When analysing the immediate and long-term effects of 7 weeks of exercise training on COPD, some scholars found that FVC% started to increase at the end of exercise and continued to increase until 52 weeks post exercise training [17]. Other studies also suggest that there is a “post-exercise effect” that can be maintained after exercise cessation [4, 18]. This phenomenon may be related to the relief of airway inflammation [19] and the decrease of acute COPD exacerbations [20]. Most studies have compared efficacy at the completion of ET and RT, while there is a lack of direct comparisons of the long-term post-exercise efficacy between the two exercise regimens in the future.

The aim of our prospective cohort study was to investigate the efficacy of endurance training versus resistance training, both of which were combined with standard medication for patients with stable COPD. Clinically relevant outcomes, such as dyspnea, exercise capacity, quality of life, and mental status (anxiety and depression subscales), were assessed at baseline and at the end of the program. All participants who completed the 12-week of a supervised training program will be followed up to 12 months. The evolution in the primary outcomes (exercise capacity, quality of life) and secondary outcomes (dyspnea, mental status) were re-evaluated at 6 and 12-month follow-up. A subgroup analysis was performed to investigate the effects of exercise training for patients with different levels of disease severity.

## Methods

### Study design

This was a multicenter, prospective, randomized clinical trial to compare the potential benefits of ET with RT in stable COPD patients. The study was approved by the ethics committee of Huadong Hospital and complied with the Declaration of Helsinki. The study was registered in Chinese Clinical Trial Registry under the number (ChiCTR-INR-16009892) and the date of first registration was 17/Nov/2016.

From November 2017 to June 2019, patients with clinically stable COPD in GOLD (Global Initiative for Chronic Obstructive Lung Disease) stage I to I were routinely recruited. Eligibility criteria for participants were forced expiratory volume in 1 s (FEV1)/forced vital capacity (FVC) ratio < 70%, FEV1 < 80% of predicted, resting arterial oxygenation > 90% and age between 40 and 80 years. Exclusion criteria consisted of the following: 1. cardiovascular problem (such as NYHA class IV, ACS), 2. diagnosed psychiatric or cognitive disorders, 3. contraindications to exercise activities (progressive neuromuscular diseases, severe orthopedic problems), 4. resting arterial PaO2 < 60 mmHg and PaCO2 > 55 mmHg, and 5. prior inclusion in a rehabilitation program (< 1 year). Verbal and written informed consent were obtained from all subjects before enrolment.

Subjects were randomly assigned to one of the following three groups with a computer-generated random sequence list: Group 1 received medical treatments (MT), Group 2 underwent an endurance exercise regimen with a combination of medical treatments (MT + ET), Group 3 was administered resistance training in conjunction with medical treatments (MT + RT). Patients in the three groups received standard individualised medical treatment according to the GOLD guidelines with the following drug regimen: 1. patients in the GOLD A group mainly inhaled tiotropium bromide, 2. inhaled tiotropium bromide or tiotropium bromide plus a long-acting β2 agonist was the main treatment for patients in the GOLD B group, 3. patients in the GOLD C group primarily inhaled tiotropium bromide with a long-acting β2 agonist, either alone or in combination with inhaled corticosteroids, 4. patients in the GOLD D group received inhalation of tiotropium bromide in addition to a long-acting β2 agonist and inhaled corticosteroids. The ABCD assessment was carried out using the COPD assessment tool outlined in the GOLD guidelines.

Assignment to intervention groups was concealed by a member of the staff who was not involved in any other aspect of the trial. Baseline clinical characteristics and history were taken prior to the initiation of the trial. The frequency of exacerbations was gathered by direct questioning of participants.

### Intervention

The exercise prescription was individualized and followed guidelines from the ATS/ERS [21]. Patients took part in either endurance or resistance training with therapists in groups of three to five individuals, three times per week for a duration of 12 weeks. Before exercise training, patients were taught by certified and experienced physiotherapists. All therapists were familiarized with the exercise regimen before the study and were given a treatment manual. All training sessions were completed at the hospital and were supervised to ensure safety, compliance and progression of training intensity. Patients performed a general warm-up before each training session that involved upper and lower limbs, trunk, stretching, and breathing exercises.

#### Endurance training

This training regimen comprised a series of aerobic exercises that were modified to integrate upper and lower body workouts. First, the endurance training session was performed on a treadmill for 20 min. The patient started walking at a speed of 1–2 miles per hour. If the patient did not experience intolerable dyspnea (Borg rating of breathlessness of 5), the speed was gradually increased to reach the target intensity. Individualized exercise intensity was adapted based on the calculated training heart rate (HR), which was more than 60% of the maximum predicted HR (208–0.7 × age) [22]. After five minutes of active recovery, the patients performed an additional free weightlifting exercise using both upper limbs to target the upper limb and trunk muscles, including the biceps brachii, pectoralis major, and latissimus dorsi. The exercise consisted of 3 sets of 30 repetitions of free lifting with a 1-min rest interval between sets. The initial load was 0.5 kg and was subsequently increased to 1.0–1.5 kg for each arm, totalling 2–3 kg [23, 24].

#### Resistance training

Resistance training was followed by the 1-repetition maximum (1RM) assessment so that further adjustments to the intensity could be made [25]. Prior to training, patients were instructed in the correct use of the resistance machines. RT consisted of 4 strength exercises of the major upper and lower body muscle groups (chest press, rowing, leg press, and leg extension). The load was initially set at 30% of 1RM and increased to 70–85% of 1RM. Once the patient was able to complete a set of 12 repetitions, the resistance (weight) was increased by 5–10%, which should not result in significant muscle fatigue or a decrease in the repeatability of the exercise. Each exercise included 5 sets with a 20-s break between sets and a 60-s break between exercises.

Patients in both exercise groups were instructed to only perform the exercises assigned to their group and to refrain from making any significant lifestyle or home exercise during the study period. In addition, patient education included standardized medical treatment for COPD, information on the health benefits of exercise, and guidelines for physical activity safety. Exercise adherence was quantified as the percentage of supervised exercise sessions completed. This was calculated by dividing the number of attended supervised exercise sessions by the number of prescribed supervised exercise sessions. The therapists recorded exercise adherence data at every supervised session.

In order to motivate the patients, the strategies were as follows: 1. Create an exercise training schedule and arrange a weekly appointment with the patient; 2. Contact patients in advance by phone to remind them of their exercise training schedule. The schedule could be brought forward or delayed by 1 or 2 days depending on the patient's condition; 3. The therapist provided positive feedback and commended the patients for their efforts in the exercise; 4. During the follow-up period, the patients were contacted in advance by phone according to the schedule and asked to come to the hospital for follow-up visits.

### Outcomes

All participants were tested at baseline and at the end of the 12-week intervention period (T0 and T3). Potential benefits were reassessed at follow-up 6 and 12 months later (T6 and T12). Minimal clinically important difference (MCID) was applied to evaluate the results. The MCID settings were obtained from a literature review.

1. ***Primary outcomes*** **:** Exercise capacity was assessed with the 3-min walking test (3MWT), where capacity was determined by the total distance walked on a flat surface (hospital hallway) in a period of 3 min. Health-related quality of life was measured using St George’s Respiratory Questionnaire (SGRQ), with a score difference > 4 units considered clinically significant (MCID) [26].
2. ***Secondary outcomes*** **:** Pulmonary function was measured using spirometry including FEV1 and FVC. To evaluate dyspnea symptoms, the Modified Medical Research Council (mMRC) scale and COPD Assessment Test (CAT) were calculated using the standard procedures for these questionnaires. MCID was -0.5 [27] and -2.0 [28] for the two assessments, respectively. Anxiety and depression were evaluated using the Hospital Anxiety and Depression Scale (HAD-A and HAD-D), and MCID was -1.3 and -1.5 for the two scales, respectively [28].

All the tests and questionnaires were completed in hospitals. The researchers furnished explanatory notes to the patients before they filled out the questionnaires. They only responded to patients' queries during the process of completion without offering recommendations or drawing conclusions on patient preferences.

### Sample size

The sample size was determined based on the minimum clinically important difference (MCID) of the primary outcome. A literature review revealed no relevant references to determine the MCID for the primary outcome of 3MWD. However, according to the literature review, the MCID for SGRQ is 4 [26]. To account for potential differences among participating hospitals in this multi-center study, we made appropriate adjustments to the sample size calculation to determine the number of participants in each center. This study was conducted across four hospitals, two general and two rehabilitation hospitals. The intraclass correlation coefficient (ICC) for center effects was taken into consideration, with the ICC being 0.05 for general hospitals and 0.1 for rehabilitation hospitals. Participants in each center were randomly assigned to one of the three treatment groups using a computer-generated random sequence list. Assuming a dropout rate of 25%, a total of 460 patients were required to be recruited to detect a 4-point difference in the primary outcome of SGRQ in the trial with a power of 80% and a significance level of 0.05 (two-tailed).

### Statistical analyses

All the data were blinded to allocation until analyses were completed. Data were analyzed using the SPSS software package (SPSS, Chicago, IL, USA), version 20. Statistical analysis was performed for both the intention-to-treat (ITT) population and per-protocol (PP) population. Baseline differences between groups were compared using a One-Way between-subjects analysis of variance (ANOVA) for continuous variables and chi-squared tests to compare proportions. A One-Way repeated-measures ANOVA was used to evaluate the difference in variables over time (from baseline to the 3rd, 6th and 12th month) within the group, followed by Bonferroni correction to identify significant differences between each time point with baseline. Change of outcomes at three time points (results at 3rd, 6th and 12th month minus baseline values) were calculated for comparison between different treatment groups. And a Two-Way mixed ANOVA was used with Bonferroni correction applied for multiple comparisons. The dependent variable was the change from baseline, while the independent variables were treatment and time, with site serving as covariates. The treatment × time interaction was tested first. If not significant, the treatment main effect was tested next. If significant, between-group differences at each time point were tested. Data was expressed as mean ± standard error of the mean. A *p* value of < 0.05 was considered to be statistically significant.

## Results

**Fig. 1:**
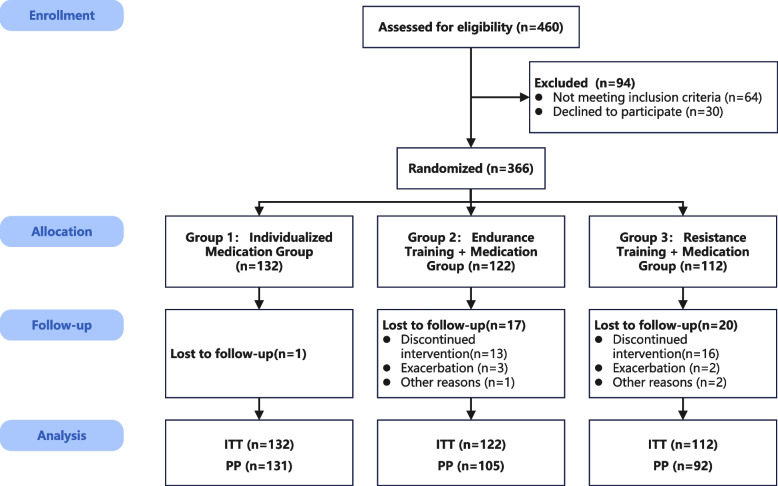
Flowchart of patient enrolment. A total of 460 patients were screened for randomisation. 64 patients were not eligible, and 30 patients declined to participate. 38 patients were lost during follow-up. In Group 1, 1 patient discontinued follow-up. In Group 2, 17 patients were lost during follow-up (patients discontinued intervention: 13; exacerbation: 3; other reasons: 1). In Group 3, 20 patients were lost during follow-up (patients discontinued intervention: 16; exacerbation: 2; other reasons: 2). ITT, intention-to-treat; PP, per-protocol

After verification of the enrolled criteria, a total of 366 patients (ITT population) were eligible to participate and randomly allocated into three groups. The detailed flow chart is outlined in Fig. 1. There were 38 drop-outs in three groups and no serious adverse events were reported. Finally, 328 patients completed the follow-up evaluations (PP population). Overall mean adherence to the supervised exercise sessions was 72% (SD = 21%). The demographics of the participants are presented in Table 1. On some variables at baseline, such as lung function and clinical status, Group 1 (MT) was not comparable to the other two exercise groups. The two exercise intervention groups were well matched for most variables at baseline, although there was a significant difference between the ET and RT groups in terms of CAT score and 3MWD.

**Table 1:** Demographic and baseline clinical characteristics of the subjects

**Variables**	** MT (*n*=132)**	** MT + ET (*n*=122)**	** MT + RT (*n*=112)**	***P*** ** -value**
**Age (yrs)**	73.75 ± 0.63	75.02 ± 0.87	74.05 ± 0.48	0.37
**Sex (% men)**	109 (82%)	72 (59%)	70 (63 %)	**<0.01**
**BMI (kg/m2)**	22.76 ± 0.50	22.57 ± 0.32	22.48 ± 0.74	0.68
**Smoking, n (%)**	99 (75%)	61 (50%)	75 (67%)	0.05
**PaO2 (mmHg)**	79.05 ± 0.50	72.0 ± 0.75	76.15 ± 0.66	0.12
**PaCO2 (mmHg)**	46.10 ± 0.56	47.23 ± 0.53	47.08 ± 0.60	0.36
**FEV1 (L)**	1.25 ± 0.05	1.80 ± 0.05	1.75 ± 0.05	**<0.05**
**FVC (L) **	2.11 ± 0.05	2.85 ± 0.05	2.87 ± 0.06	**<0.05**
**FEV1/FVC**	0.59 ± 0.04	0.63 ± 0.04	0.61 ± 0.04	0.10
**GOLD group, n (%)**				
** A and B**	45 (34%)	64 (52%)	59 (53%)	
** C and D**	87 (66%)	58 (48%)	53 (47%)	
**SGRQ**	21.20 ± 0.67	19.46 ± 0.75	18.55 ± 0.80	0.35
**3MWD (m)**	155.36 ± 3.21	108.32 ± 4.06	130.24 ± 3.67	**<0.01**
**CAT**	16.60 ± 0.52	13.74 ± 0.45	13.02 ± 0.51	**<0.05**
**mMRC**	2.18 ± 0.05	1.85 ± 0.06	1.21 ± 0.06	0.32
**HAD-A**	6.03 ± 0.20	6.37 ± 0.36	6.23 ± 0.17	0.58
**HAD-D**	6.61 ± 0.26	9.35 ± 0.31	8.67 ± 0.35	**<0.01**

*MT* medical treatment, *MT + ET* medical treatment combined with endurance training, *MT + RT* medical treatment combined with resistance training, *BMI* Body mass index, *PaO2* Arterial oxygen partial pressure, *PaCO2* Arterial carbon dioxide partial pressure, *FEV1* forced expiratory volume in 1 s, *FVC* forced vital capacity, *GOLD* Global Initiative for Chronic Obstructive Lung Disease, *CAT* COPD assessment test, *mMRC* Modified Medical Research Council, *SGRQ* St George’s Respiratory Questionnaire, *3MWD* 3min walking distance, *HADS* Hospital Anxiety and Depression Scale (D = depression subscale; A = anxiety subscale). Data are presented as number (%) of patients or mean ± standard error of the mean (SEM)

**Fig. 2:**
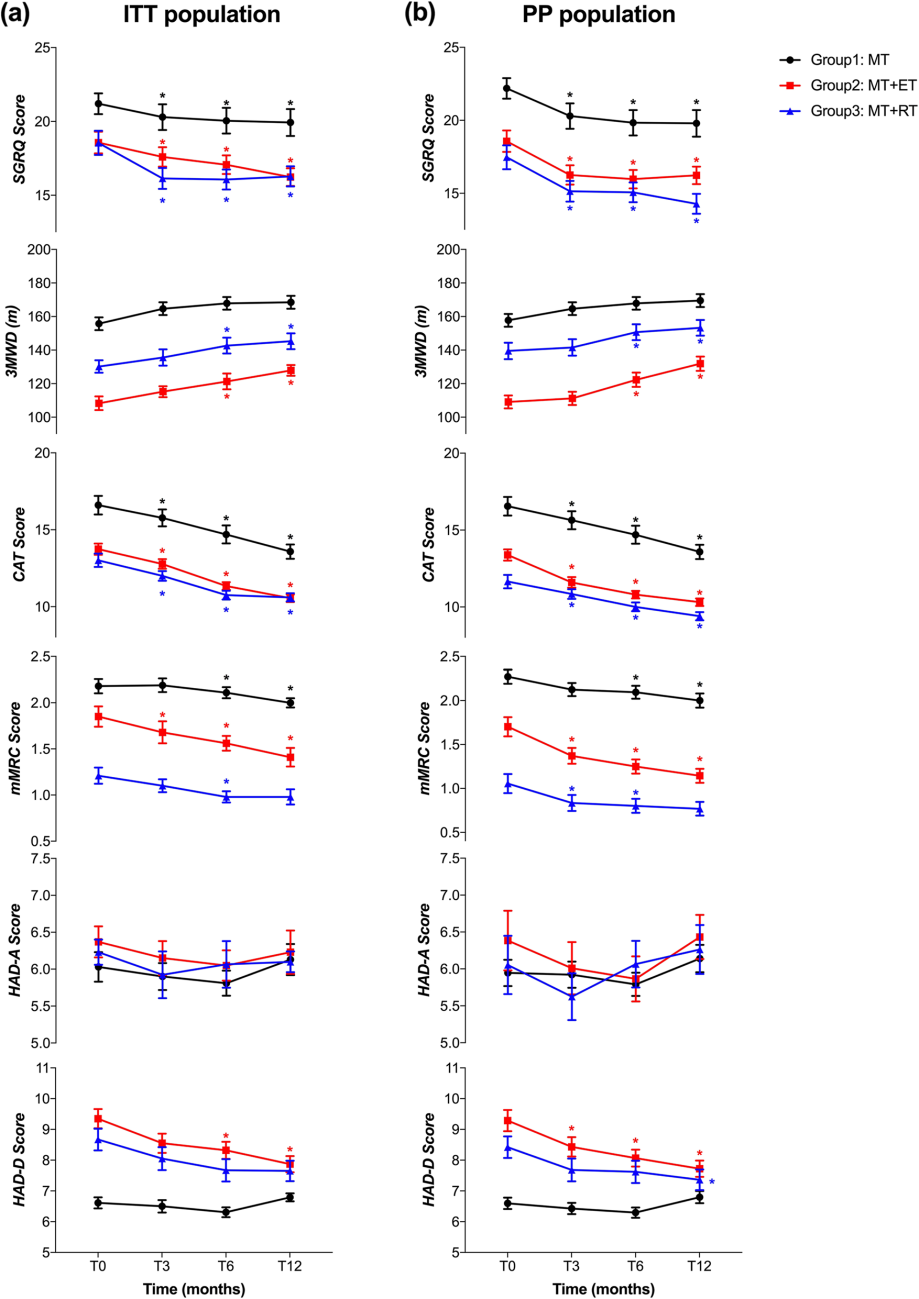
Outcomes at baseline (T0), at the end of the intervention (T3), and at follow-ups of 6-month (T6) and 12-month (T12) in the ITT (left panel) and the PP population (right panel) are shown. Group 1, medical treatment (MT); Group 2, medical treatment combined with endurance training (MT + ET); Group 3, medical treatment combined with resistance training (MT + RT). All values are shown as mean ± SEM. * Repeated measures between the baseline and the indicated time points within groups: *P* < 0.05

To better evaluate the continuous effects of exercise, Fig. 2 displayed the primary and secondary outcomes of each group at various time points. The within-group efficacy of the different interventions was assessed using a self-contrast method (one-way repeated-measures ANOVA). In the ITT analysis shown in Fig. 2a, both exercise intervention arms demonstrated significant reductions in primary outcomes and most secondary outcomes immediately after exercise regimens and/or at the subsequent follow-up time points. The results of the PP analysis were also similar, as shown in Fig. 2b.

**Fig. 3:**
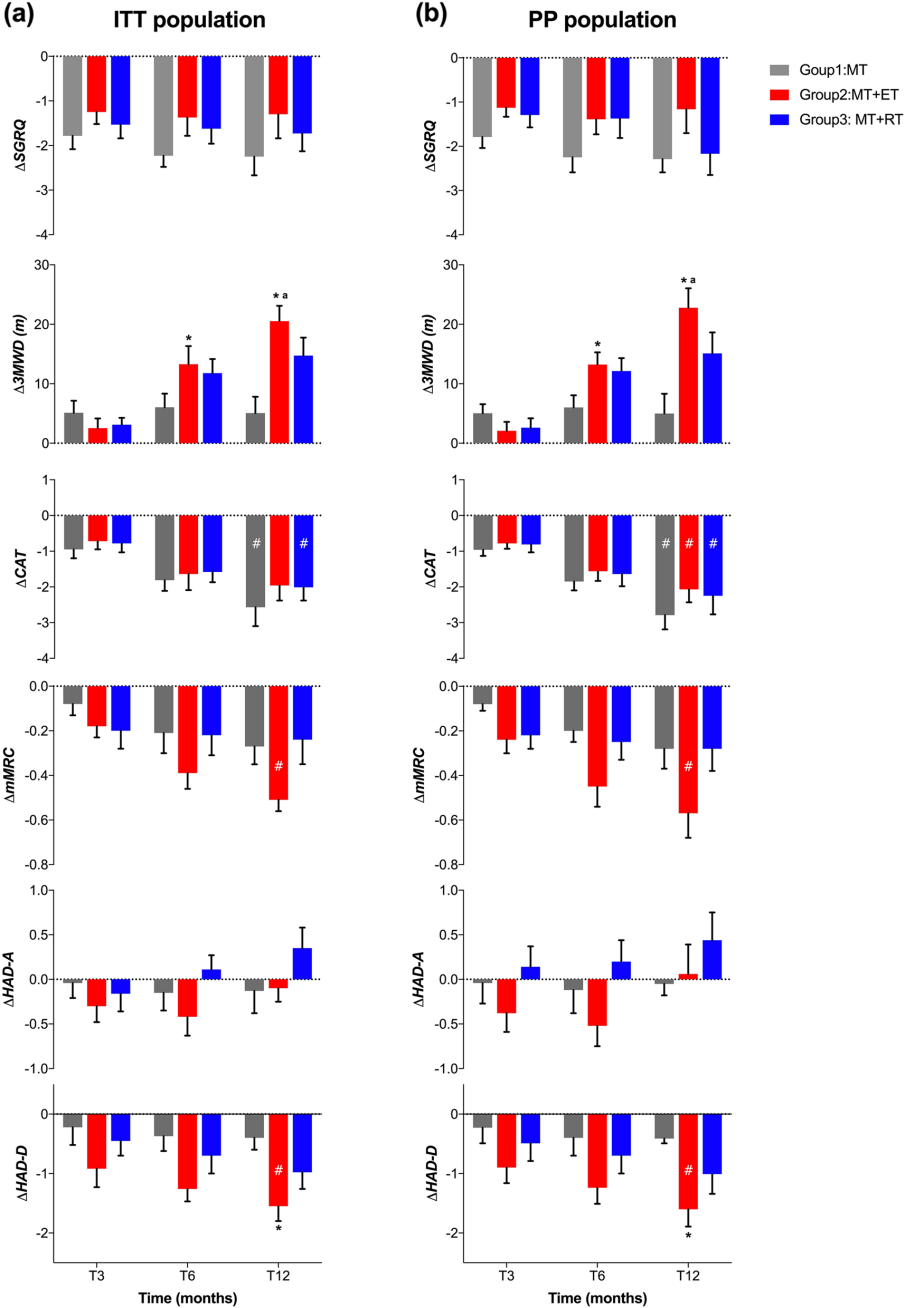
Comparison of changes in outcomes from baseline to each time point between the three treatment groups in the ITT (left panel) and the PP population (right panel). Group 1, medical treatment (MT); Group 2, medical treatment combined with endurance training (MT + ET); Group 3, medical treatment combined with resistance training (MT + RT). Δ: value changes between the baseline and different time points of follow-up. T3, at the end of programs; T6 and T12, at 6 and 12-month follow-up. * Different from the MT group: *P* < 0.05. ^**a**^Difference between the exercise training groups: *P* < 0.05. **#**, the change in value exceeded the minimum clinically important difference (MCID) for the outcome

As the study aimed to compare the effects of different treatments, the following section details the results of the between-group effects. It is worth noting that some variables showed differences in baseline levels among the three groups as mentioned above. Therefore, for each outcome, we calculated the change from baseline (Δ-outcome) at follow-up time points and compared the effects of different interventions using a two-way mixed ANOVA, as presented in Table 2 and Fig. 3.

**Table 2:** Changes in outcomes from baseline to time points of follow-up by ITT and PP analysis

		**ITT population**			**PP population**		
**Variables**	**Treatment**	**Time**			**Time**		
		**T3**	**T6**	**T12**	**T3**	**T6**	**T12**
***Primary outcomes***							
** ΔSGRQ**	**MT**	-1.78 ± 0.30	-2.23 ± 0.25	-2.25 ± 0.42	-1.79 ± 0.25	-2.25 ± 0.34	-2.29 ± 0.30
	**MT + ET**	-1.25 ± 0.27	-1.37 ± 0.41	-1.30 ± 0.55	-1.13 ± 0.20	-1.39 ± 0.34	-1.16 ± 0.54
	**MT + RT**	-1.53 ± 0.31	-1.62 ± 0.34	-1.73 ± 0.40	-1.29 ± 0.28	-1.37 ± 0.44	-2.17 ± 0.48
	**MT**	5.12 ± 2.01	6.05 ± 2.28	5.07 ± 2.74	5.05 ± 1.51	6.00 ± 2.07	4.99 ± 3.33
** Δ3MWD(m)**	**MT + ET**	2.55 ± 1.61	13.28 ± 3.07*	20.53 ± 2.58* ^**a**^	2.09 ± 1.49	13.23 ± 2.05*	22.78 ± 3.29* ^**a**^
	**MT + RT**	3.12 ± 1.15	11.79 ± 2.38	14.74 ± 3.02	2.60 ± 1.59	12.13 ± 2.19	15.12 ± 3.50
***Secondary outcomes***							
** ΔCAT**	**MT**	-0.95 ± 0.25	-1.81 ± 0.30	-2.57 ± 0.53^**#**^	-0.96 ± 0.20	-1.85 ± 0.28	-2.79 ± 0.40^**#**^
	**MT + ET**	-0.72 ± 0.23	-1.64 ± 0.45	-1.96 ± 0.42	-0.78 ± 0.15	-1.56 ± 0.27	-2.07 ± 0.36^**#**^
	**MT + RT**	-0.78 ± 0.25	-1.58 ± 0.29	-2.01 ± 0.37^**#**^	-0.81 ± 0.22	-1.64 ±0.34	-2.25 ± 0.52^**#**^
	**MT**	-0.08 ± 0.05	-0.21 ± 0.09	-0.27 ± 0.08	-0.08 ± 0.03	-0.20 ± 0.08	-0.28 ± 0.10
** ΔmMRC**	**MT + ET**	-0.18 ± 0.05	-0.39 ± 0.07	-0.51 ± 0.05^**#**^	-0.24 ± 0.06	-0.45 ± 0.08	-0.57 ± 0.09^**#**^
	**MT + RT**	-0.20 ± 0.08	-0.22 ± 0.09	-0.24 ± 0.11	-0.22 ± 0.06	-0.25 ± 0.08	-0.28 ± 0.10
	**MT**	0.04 ± 0.17	-0.15 ± 0.20	-0.13 ± 0.25	0.04 ± 0.23	-0.12 ± 0.26	-0.11 ± 0.32
** ΔHAD-A**	**MT + ET**	-0.30 ± 0.18	-0.42 ± 0.21	-0.10 ± 0.15	-0.38 ± 0.21	-0.52 ± 0.23	0.05 ± 0.21
	**MT + RT**	-0.16 ± 0.20	0.11 ± 0.16	0.35 ± 0.23	0.14 ± 0.23	0.20 ± 0.25	0.43 ± 0.31
	**MT**	-0.22 ± 0.30	-0.37 ± 0.25	-0.40 ± 0.20	-0.23 ± 0.26	-0.40 ± 0.30	-0.41 ± 0.08
** ΔHAD-D**	**MT + ET**	-0.92 ± 0.31	-1.26 ± 0.21	-1.55 ± 0.25* ^**#**^	-0.90 ± 0.26	-1.24 ± 0.27	-1.60 ± 0.29* ^**#**^
	**MT + RT**	-0.45 ± 0.25	-0.70 ± 0.30	-0.98 ± 0.28	-0.49 ± 0.30	-0.70 ± 0.29	-1.01 ± 0.32
	**MT**	0.015 ± 0.03	0.038 ± 0.03	0.042 ± 0.03	0.015 ± 0.02	0.039 ± 0.02	0.045 ± 0.03
** ΔFEV1(L)**	**MT + ET**	0.021 ± 0.02	0.065 ± 0.03	0.171 ± 0.03*	0.020 ± 0.02	0.069 ± 0.02	0.176 ± 0.03*
	**MT + RT**	0.025 ± 0.03	0.044 ± 0.02	0.090 ± 0.02	0.022 ± 0.02	0.043 ± 0.02	0.089 ± 0.03
	**MT**	0.014 ± 0.02	0.024 ± 0.03	0.037 ± 0.01	0.015 ± 0.02	0.025 ± 0.02	0.037 ± 0.03
** ΔFVC(L)**	**MT + ET**	0.020 ± 0.03	0.051 ± 0.02	0.158 ± 0.02*	0.019 ± 0.02	0.047 ± 0.02	0.169 ± 0.03*
	**MT + RT**	0.022 ± 0.02	0.055 ± 0.03	0.145 ± 0.02*	0.020 ± 0.02	0.059 ± 0.02	0.152 ± 0.02*

Δ, value changes between the baseline and different time points of follow-up

*T3* at the end of programs, *T6 and T12* at 6 and 12-month follow-up, *SGRQ* St George’s Respiratory Questionnaire, *3MWD* 3min walking distance, *CAT* COPD assessment test, *mMRC* Modified Medical Research Council, *HADS* Hospital Anxiety and Depression Scale (D = depression subscale; A = anxiety subscale), *ITT* Intention-to-treat, *PP* per-protocol

Data are presented as mean ± SEM. ^**#**^, the change in value exceeded the minimum clinically important difference (MCID) for the outcome

*Different from the MT group: *P* < 0.05. ^a^Difference between the exercise training groups: *P *< 0.05

### Effects on primary outcomes

#### SGRQ

Although the medical treatment and both exercise regimens showed significant reductions in SGRQ scores, there was no difference in the change of SGRQ among the three groups during the follow-up time points in both the ITT and PP analysis (refer to Table 1, Fig. 3a and b).

##### 3MWD

The comparison of the changes in the 3MWD between the two groups is shown in Table 2. No significant differences were observed between groups after initial 3 months of intervention. At 6 and 12-month of follow-up, the increase in 3MWD after ET was significantly greater than that after MT in the ITT populations. In the PP populations, it was also observed that ET had a higher priority than MT at the same time points. Furthermore, the improvement in 3MWD after ET was significantly better than that after RT at the 12-month follow-up in both the ITT and PP populations (ITT: Δ3MWD in ET vs Δ3MWD in RT = 5.53 m, 95% confidence interval: 0.87 to 13.84 m, *P* = 0.03) (PP: Δ3MWD in ET vs Δ3MWD in RT = 7.67 m, 95% confidence interval: 0.93 to 16.27 m, *P* = 0.04) (Table 2, Fig. 3a and b).

#### Effects on secondary outcomes

##### CAT and mMRC

Although the CAT score exhibited a continuous decrease following medical treatment and both exercise regimens in the ITT and PP populations, there were no significant differences in score changes between the MT, ET or RT regimens at any time point when comparing their effects between groups (Table 2 and Fig. 3).

Similar changes were also noted with dyspnea symptoms measured by mMRC. Although the decrease in mMRC score exceeded the MCID (-0.5) at the 12-month mark after ET in both the ITT and PP populations, there were no significant differences in the change of mMRC score among the three groups at any time point (ΔmMRC in ET, ITT: -0.51 ± 0.05, PP: -0.57 ± 0.09) (Table 2 and Fig. 3).

#### HAD-A and HAD-D

Although a reduction in HAD-A score was observed after ET in the ITT and PP populations, there were no significant differences in the comparison of HAD-A score changes among the three groups at each time point (Table 1 and Fig. 3).

With regard to the change in HAD-D score during follow-up, the ET group achieved a significant improvement over the MT group at the 12-month time point in both ITT and PP populations (Fig. 3a and b). Although there was no significant difference in the score change between ET and RT, it appears that endurance training could effectively alleviate depression, with the change exceeding MCID (-1.5) at the end of the study (ΔHAD-D in ET at 12-month: -1.55 ± 0.25in ITT, -1.60 ± 0.29 in PP) (Table 1 and Fig. 3).

#### Lung funSction

There was a trend towards improvement in lung function (FVC and FEV1) after ET and RT over the follow-up period. In the ITT analysis, patients undergoing ET achieved greater improvement in FEV1 and FVC at 12-month than with MT alone. Similarly, patients undergoing RT showed better FVC improvement at 12-month compared to those who received MT alone. The results of the PP analysis were consistent with those of the ITT analysis. However, no significant difference was observed between ET and RT at each time point (Table 2).

#### Subgroup analysis

**Fig. 4:**
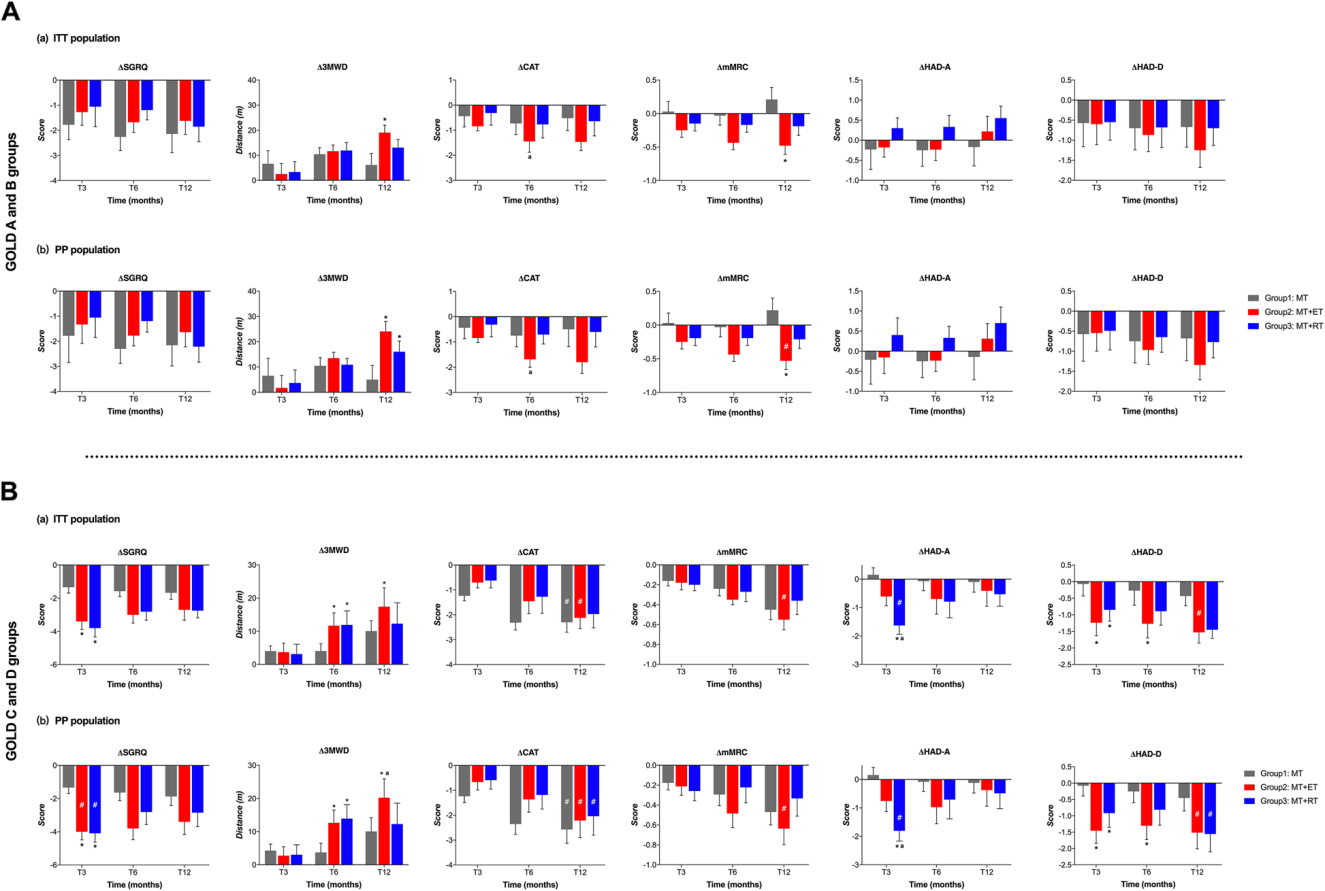
Comparison of changes in outcomes from baseline to different time points between treatments with patients being stratified into GOLD A + B and GOLD C + D groups in the ITT and the PP population. Group 1, medical treatment (MT); Group 2, medical treatment combined with endurance training (MT + ET); Group 3, medical treatment combined with resistance training (MT + RT). Δ: value changes between baseline and different time points of follow-up. T3, at the end of programs; T6 and T12, at 6 and 12-month follow-up. * Different from the MT group: *P* < 0.05. ^a^Difference between the exercise training groups: *P* < 0.05. **#**, the change in value exceeded the minimum clinically important difference (MCID) for the outcome.

Patients were further divided into the GOLD A + B group and GOLD C + D group according to the COPD assessment tool offered by GOLD guidelines. Changes in outcomes were compared among the three groups at each follow-up time point (Fig. 4).

#### SGRQ and 3MWD

For patients in GOLD A + B group, there was no difference in SGRQ changes between the three groups in both ITT and PP populations (Fig. 4A-a and A-b). However, patients in the GOLD C + D group who underwent either type of exercise treatment showed significantly greater changes in SGRQ scores compared to those in the MT group upon completion of the exercise (T3) in the ITT population (Fig. 4B-a). The PP population also showed similar results, meeting or exceeding the MCID (-4.0) in the PP analysis (ΔSGRQ at T3: ET = -4.0 ± 0.48, RT = -4.1 ± 0.54), which was not observed in the ITT analysis (Fig. 4B-b). Additionally, there was no significant difference in the SGRQ score changes between the two exercise treatments administered to these patients.

In the ITT population, patients classified as GOLD A + B who underwent ET showed a greater increase in 3MWD at 12-month compared to those who received MT alone. However, those who received RT did not show a significant difference (Fig. 4A-a). In the PP population, both types of exercise training resulted in a greater improvement in 3MWD compared to medical therapy alone at 12-month (Fig. 4A-b). There was no significant difference between endurance training and resistance training. For patients in the GOLD C + D group, the increase in 3MWD was greater than that of MT after either ET or RT in the ITT population at the 6-month follow-up, and the advantage of ET persisted at 12-month (Fig. 4B-a). Additionally, the improvement in 3MWD after ET was significantly better than that after RT in the PP population (Δ3MWD in ET vs RT at 12-month: 20.24 ± 5.70 vs 12.30 ± 6.29 m, *P* < 0.01) (Fig. 4B-b).

#### CAT and mMRC

Both CAT and mMRC scores decreased gradually after exercise in patients with different disease severities. Patients stratified in the GOLD A + B group showed greater improvement in CAT score at the 6-month follow-up after ET than RT (ΔCAT in ET vs RT at T6, ITT: -1.45 ± 0.43 vs -0.77 ± 0.54, PP: -1.69 ± 0.31 vs -0.71 ± 0.38, both *P* < 0.05) (Fig. 4A-a, A-b). Meanwhile, in the GOLD A + B group, patients who underwent ET showed greater improvement in mMRC score changes at the 12-month follow-up compared to those who underwent MT in both the ITT and PP populations, which exceeded the MCID (-0.5) in the PP population (ΔmMRC at T12 in ET = -0.53 ± 0.12 in PP) (Fig. 4A-b). For patients stratified into GOLD C + D groups, both CAT and mMRC scores exceeded the MCID after ET according to ITT analysis, but changes in these scores were not significantly different between treatments (Fig. 4B-a). Similar results were also observed in the PP populations (Fig. 4B-b).

#### HAD-A and HAD-D

In the subgroup analysis, the anxiety scale showed no change in the GOLD A + B group after either ET or RT (Fig. 4A). Nevertheless, we found that patients in the GOLD C + D group experienced greater relief from anxiety after completing RT compared to ET and MT as shown in Fig. 4B. At the 3-month time point, RT demonstrated a significantly greater change in HAD-A scores compared to ET in both the ITT analysis (ΔHAD-A: RT = -1.63 ± 0.31 vs ET = -0.61 ± 0.33, *p* < 0.01) and the PP analysis (ΔHAD-A: RT = -1.80 ± 0.36 vs ET = -0.75 ± 0.37, *p* < 0.01). And the changes in HAD-A score at this time point also exceeded MCID (-1.3) (Fig. 4B-a, b). In the subgroup analysis of HAD-D, no significant differences between treatments were observed during follow-up in the GOLD A + B group (Fig. 4A). However, in the ITT analysis, patients in the GOLD C + D group reported greater relief from depression immediately after completing ET and RT compared to MT. This advantage of ET over MT was sustained for up to 6 months of follow-up (Fig. 4B-a). In the PP population, patients stratified in the GOLD C + D group presented similar results in HAD-D score changes as those shown in the ITT populations (Fig. 4B-b). At the 12-month mark, the improvement in HAD-D after the exercise regimens exceeded the MCID (-1.5) in both the ITT and PP analyses, but did not show a statistically significant difference compared to MT. In addition, there was no discernible difference between the two exercise training regimens in alleviating HAD-D at the specified time points.

#### Adverse events during the intervention

During the intervention period, a small proportion of the patients reported adverse events during or after training sessions. In the ET group, 45 out of 122 (36.9%) patients reported adverse events. These comprised muscle soreness in the lower limbs (*n* = 19), increased short-term pain in the knees (*n* = 18), discomfort due to hypertension (*n* = 1) and shortness of breath (*n* = 7). In the RT group, 40 out of 112 participants (35.7%) reported adverse events. This included muscle soreness in the lower limbs (*n* = 18), increased short-term pain in the knees (*n* = 9) and shoulders (*n* = 8), as well as discomfort due to nausea (*n* = 1) and shortness of breath (*n* = 4). In total, 29 patients discontinued the exercise intervention due to adverse events.

## Discussion

Accumulating evidence indicates that skeletal or respiratory muscle dysfunction contributes to impairment of COPD, which can be counteracted by exercise training. Our study compared the effects of two commonly used exercise regimens, endurance training and resistance training, and found that both exercise regimens improved quality of life, exercise capacity, dyspnea and psychological state in COPD patients. Although no difference was observed after the initial 3 months of intervention, improved exercise capacity and reduced depression were observed after ET compared to MT, as evaluated by 3MWD and HAD-D scores, at the 6 and 12-month follow-up. After the cessation of exercise, a slight improvement in 3MWD compared to RT was observed during the follow-up period for ET. Subgroup analyses indicate that the effects of ET and RT were comparable in patients with different conditions on most of the indicators assessed, but there were differences in efficacy for 3MWD, CAT and HAD-A scores.

It was observed that exercise capacity, as measured by 3MWD, was sustained for a long time after exercise training. Additionally, 3MWD showed greater improvement after ET than MT during the follow-up period after exercise cessation. This result is consistent with studies which agree that combining any exercise modality with conventional medical therapy could produce more lasting benefits in COPD patients [29]. It is worth noting that in this study, neither endurance nor resistance training showed superiority over standard medical treatment after the first three months, which is in contrast to the established literatures [29, 30]. The potential reasons are as follows. Although we aimed to include diverse patient types in our study design, during implementation we found that patients with better exercise capacity and less severe symptoms were more willing to engage in exercise training. Therefore, compared to those reported in previous literature, our recruited patients had relatively lower baseline scores for both primary and secondary outcomes. One study showed that patient's baseline condition may influence or limit the extent of improvement when assessing outcomes after exercise training [27]. In other words, patients with poorer baseline conditions may derive greater benefit from exercise training. The patients in our study generally exhibited a higher quality of life and milder symptoms prior to intervention compared to those in other studies. This may have contributed to the absence of a significant advantage over medical treatment upon completion of exercise training. Therefore, the patients were stratified according to the GOLD assessment tool. Subgroup analyses showed a more significant improvement in the quality of life among patients with more severe conditions after exercise training. Additionally, the selection of follow-up time points may impact the observation of results, as some effects may require more time to become apparent. Therefore, we extended the follow-up period and observed that exercise training was more effective than medical treatment in terms of sustained efficacy on 3MWD.

Our data showed that ET was more effective than RT in increasing 3MWD for up to 12 months after exercise. This could be explained by the improved aerobic metabolism phenotype, thus leading to a reduced ventilatory demand and potentially attenuating dyspnea [31]. Although resistance training can improve aerobic metabolic capacity, such as an increase in maximal oxygen uptake (VO2max), our previous study using a cardiopulmonary exercise test (CPET) showed that endurance training resulted in a significant decrease of ventilatory demand (evaluated by the maximum ventilation volume, VEmax) and greater improvement in aerobic metabolism (VO2max and VO2/kg) compared to resistance training [32]. This suggests that endurance training may be more beneficial for improving aerobic metabolic capacity. During the follow-up after both ET and RT, we observed a continuous increase in 3MWD. This finding is consistent with many other studies before [8, 33]. However, a difference in the change of 3MWD between ET and RT was only observed at 12 months, not at 3 or 6 months. Berry's study showed similar results to ours, indicating no significant difference between ET and RT in walking performance (6MWD) after 12 weeks of exercise training [8]. However, no further observations were conducted in the subsequent 6 or 12 months. The observation period of our study extended beyond the end of exercise, and the sustained efficacy suggests that exercise training has a lasting effect. As previously mentioned, ET has been shown to be more effective than RT in improving aerobic metabolic capacity. Therefore, a longer observation period may be necessary to detect a significant difference in efficacy between the two exercise regimens. Moreover, it is important to be cautious about concluding that ET significantly improves exercise capacity compared to RT. It is necessary to consider whether the difference in efficacy observed at 12 months was entirely attributable to the exercise itself, after 12 weeks of both exercise regimens had ceased. Although we conducted relevant quality control in our subsequent follow-up, we cannot entirely rule out the possibility that other uncontrollable factors may have influenced the results. Therefore, future studies could consider using multiple indices to assess exercise capacity, which can provide mutual support when interpreting the results. On the other hand, it is important to note that there is currently no established MCID reference range for the 3MWD. Therefore, the observed difference of 5.53 m between ET and RT at 12 months may not be clinically significant. Additionally, the study used the 3MWD for evaluation, which correlates well with the 6MWD but may have differences in diagnostic efficacy. This may have contributed to the lack of statistically significant difference observed in 3MWD changes between the ET and RT groups at 6 months.

In our study, no difference was found between ET and RT in the improvement of SGRQ. It is interesting to note that, in Berry’s study, similar improvement in walking performance (6MWD) after ET and RT was shown, while on the other hand they showed that endurance training was superior to strength training in terms of health-related quality of life assessed by the Short Form 36 (SF-36) [8]. This phenomenon can largely be attributed to different psychological and physical test instruments. The SGRQ questionnaire consists of three main domains including symptom, impact and activity, which focuses on respiratory symptoms and their impact on daily activities. The SF-36 is a generic health survey that measures overall health-related quality of life. One study found that the SGRQ demonstrated greater ability to discriminate among different levels of severity stages of COPD than the SF-36 [34]. Although no differences in the effects on SGRQ changes were found among the three treatments in all COPD patients in our study, after stratifying into different severity groups, ET and RT showed greater improvement on SGRQ than MT alone. On the other hand, the total SGRQ score was used for the evaluation between ET and RT, which would probably be different if the scores in each component were in comparison. Further research is required to determine its probability.

Subgroup analyses show that ET and RT were equally effective for patients with different disease severity. However, differences in exercise capacity (3MWD) and psychological status (HAD-D) improvement were observed among more severe patients who underwent different types of exercise regimen. According to the results of the PP analysis, patients stratified into GOLD C and D groups experienced greater long-term improvements in 3MWD with ET compared to RT. However, this difference was not observed in the ITT analysis. This discrepancy may be attributed, in part, to the fact that ITT analysis includes all randomly allocated participants, including those who may have faced challenges during the exercise regimen and dropped out. For patients who adhere to completing the exercise training, ET may have a more significant impact on improving exercise capacity. When evaluating the effects of different training modalities, it is important to consider factors such as patient integrity and treatment adherence comprehensively. In both the ITT and PP analysis, a significant improvement in HAD-A score after RT compared to ET was observed in these patients. Consistent with previous research, resistance training was considered to be an effective intervention for reducing symptoms of anxiety [35]. Meanwhile, these findings were partly consistent with those reported by Würtemberger et al., who found that people with severe disease responded better to exercise training [36]. Moreover, our study suggests that exercise regimens could be selected based on disease conditions of individual patients. For instance, patients in more severe stages with decreased exercise capacity are recommended to do endurance training to increase exercise tolerance, and patients with anxiety are advised to opt for resistance training to alleviate emotional stress. Patients with less severe COPD are advised to choose between endurance and resistance training according to their preference, or to prioritise endurance training for better symptom control. It should be noted that our study design was not powered to detect differences in these subgroups, which may explain the absence of significant results when comparing ET and RT.

Although our results showed no significant difference in the efficacy of the two types of training regimen for most outcomes, the improvement in some outcomes after either ET or RT met or exceeded the MCID. In practice, it is important to achieve clinically meaningful improvement from treatment rather than statistical significance. Our subgroup analysis stratified by disease severity showed that patients in the GOLD C and D groups achieved a clinically meaningful change in quality of life (SGRQ) after both ET and RT in the PP analysis. Consistent with our findings, Paneroni et al. confirmed that aerobic exercise training improved exercise tolerance and health-related quality of life in very severe COPD [37]. Similar results were observed for several other outcomes, suggesting that exercise training may provide greater clinical benefits for critically ill patients. Therefore, it is important to encourage patients, particularly those in the severe stage, to participate in exercise training.

When comparing the changes in absolute FEV1 and FVC, Strasser and colleagues suggested that resistance training might be a better alternative to improve lung function [38], whereas no dominance was observed in our study. Meanwhile, mild long-term increases in FVC were observed after both ET and RT, indicating that changes in lung function after exercise may be delayed. Yu et al. documented that pulmonary rehabilitation by itself does not improve lung function but may carry potential benefits for FVC [39]. Egan et al. also noted an upward trend in FVC% at three months after exercise, which was maintained until 52 weeks after cessation of exercise [17]. Additionally, we observed that the improvement in exercise capacity was maintained for a long time after exercise training, which may indirectly affect the change in lung function.

Maintaining the beneficial effects after programme completion is currently an important issue [40]. Longer durations of programs were reported to have a more favorable effect on HRQL in a meta-analysis [41]. To better promote the maintenance of benefits over time, our training prescription was 12 weeks in duration, in line with ACCP recommended guidelines [3]. On the other hand, the results may be discrepant depending on the varied evaluation time [42]. Only a limited number of studies have examined changes in outcomes both immediately after exercise training and during the follow-up period. In the present study, we extended the follow-up period and found that improvements in some parameters persisted at 12 months after a 12-week training intervention. This means that patients can still sustain clinical improvement after ceasing exercise training in a period of time. Exercise training over 12 weeks may produce up to 48 weeks of post-exercise effects.

The strengths of this study lie in its rigorous methodology. Our results have not been affected by inclusion or selection bias. The intensity setting for endurance training in our study was based on the maximum expected heart rate, which is not as objective as a cardiopulmonary exercise test, but is convenient for studies with large sample sizes [22]. Our resistance training program was well tolerated using 1RM as the standard for adjusting exercise intensity [25]. Over 70% of participants completed each program and no serious adverse events were reported. In addition, to ensure the objectivity and accuracy of the results, the blinded assessments were similar to those of previous studies [43].

Regarding the design of the exercise program, the regimen included exercising the upper and lower limb muscle groups. Endurance training exercises typically involve the use of treadmills and cycle ergometers, with a focus on the lower limb muscle groups. However, omitting upper limb muscle group training may weaken the improvement in ventilatory capacity achieved through exercise training [44]. To enhance the patient's exercise capacity and meet their practical needs, we considered that combined upper and lower limb exercise training would be more appropriate. Unsupported upper limb endurance training with dumbbells [24] is an appropriate and convenient method to integrate into our endurance training regimen. In the design of the program, the resistance training involved both the upper and lower body, and the endurance training was optimised for walking with the addition of free lifting of objects with the upper limbs. This study design excluded the influence of the range of muscle groups trained on effectiveness. This allowed for the assessment of the effects of exercise solely due to differences in training modalities.

To ensure adherence and fidelity to the exercise intervention, certain methods were employed. Prior to the trial, researchers and physiotherapists received training on delivering the trial protocol. They were provided with a detailed manual describing each exercise intervention. During the trial, regular on-site and telephone meetings were held to discuss any issues experienced and to suggest solutions.

### Study limitations

Several limitations should be addressed. In our study, patients did not reach similar levels at the baseline when they were enrolled. For instance, the medication group (MT) had a higher proportion of patients in GOLD C and D groups (66%) compared to the exercise training group (ET or RT) (47–48%). On the other hand, it is worth noting that the outcomes of the three groups were not perfectly matched at baseline. Therefore, we calculated the change in outcomes from baseline at each time point (delta-outcome) to compare the effect of different treatment programs. This approach aims to make the assessment more objective.

Another limitation of this study is the lack of assessment of muscle function. COPD patients have varying degrees of skeletal muscle dysfunction, affecting both respiratory and non-respiratory muscle groups. Age, severity of COPD, and dyspnea degree are closely associated with the loss of muscular mass and force [45, 46]. While the study focused on the effects of exercise training on various outcomes, such as quality of life and endurance, it did not include specific measurements of muscle function. Thus, the study may have missed valuable insights into the specific effects of exercise training on muscle function. Future research should consider incorporating comprehensive measures of muscle function to provide a more complete understanding of the impact of exercise training on overall physical fitness and performance.

## Conclusions

The study compared the effects of endurance training and resistance training and found no significant difference between the two exercise regimens when added to standard medication in improving quality of life, dyspnea, psychological state, or lung function among patients with stable COPD. However, endurance training exhibited a slight, statistically significant advantage over resistance training on exercise capacity at the 12-month follow-up. Nonetheless, it is important to be cautious in concluding that endurance training is superior to resistance training in terms of improving exercise capacity. Our study also sought to provide evidence to help optimize exercise programs for patients in different stages of the disease. Patients in severe stage may derive greater benefit from exercise training, particularly in improving exercise capacity through endurance training and psychological status through resistance training. Therefore, it is important to increase participation and encourage personalized exercise training in this group of patients. The combination of exercise and medication produced most of the benefits without increasing adverse events, and both interventions were found to be beneficial. More importantly, individual adherence to a rehabilitation program and personal preferences should be taken into account.

## Data Availability

The datasets in the current study are available from the corresponding author on reasonable request.

## Abbreviations

COPD: Chronic obstructive pulmonary disease
PR: Pulmonary rehabilitation
ET: Endurance training
RT: Resistance training
PaO2: Arterial oxygen partial pressure
PaCO2: Arterial carbon dioxide partial pressure
FEV1: Forced expiratory volume in 1 s
FVC: Forced vital capacity
GOLD: Global Initiative for Chronic Obstructive Lung Disease
CAT: COPD assessment test
mMRC: Modified Medical Research Council
HRQL: Health-related quality of life
SGRQ: St George’s Respiratory Questionnaire
3MWD: 3 Min walking distance
HADS: Hospital Anxiety and Depression Scale (D: depression subscale; A: anxiety subscale)
MCID: Minimal clinically important difference
CPET: Cardiopulmonary exercise test
SF-36: Short Form 36
